# Resisting the Toxic Tide: Multi-Metal Resistance of Bacteria Originating from Contaminated Šibenik Bay Sediments

**DOI:** 10.3390/microorganisms13102326

**Published:** 2025-10-08

**Authors:** Ana Ramljak, Marta Žižek, Anastazija Huđ, Goran Palijan, Mavro Lučić, Ines Petrić

**Affiliations:** 1 Ruđer Bošković Institute, 10000 Zagreb, Croatia; aramljak@irb.hr (A.R.); mzizek2@gmail.com (M.Ž.); ahudj@irb.hr (A.H.); mlucic@irb.hr (M.L.); 2 Department of Biology, Josip Juraj Strossmayer University of Osijek, 31000 Osijek, Croatia; gpalijan@biologija.unios.hr

**Keywords:** bacterial isolates, metal resistance, metal mixtures, contaminated sediments, marine ecosystems

## Abstract

In this study, 74 bacterial isolates were obtained from sediments of Šibenik Bay, which has historically been impacted by heavy metal pollution. Isolates were tested for tolerance to cadmium (Cd), cobalt (Co), chromium (Cr), copper (Cu), mercury (Hg), nickel (Ni), lead (Pb), tin (Sn), and zinc (Zn), both individually and in mixtures, and for their biofilm-forming ability. Toxicity followed the trend Hg > Sn > Zn/Cd/Cr > Co/Ni > Pb, with Cu showing resistance across different concentrations. Resistance traits were observed against all tested metals, with some isolates displaying multi-metal resistance to as many as seven metals, reflecting long-term selective pressure in the Bay. The *Bacillus* species dominated the community (48 isolates across five clusters), confirming this genus as the principal group in metal-polluted sediments. Several less-explored genera, including *Ruegeria*/*Cribrihabitans, Bhargavaea*, *Pseudoalteromonas*, and *Lysinibacillus*/*Sporosarcina*, also exhibited notable resistance traits, underscoring their potential as novel candidates for bioremediation. Eleven isolates from *Bacillus*/*Mesobacillus*/*Cytobacillus*, *Bacillus*/*Peribacillus*/*Rossellomorea*, *Bacillus*/*Pseudoalkalibacillus*/*Alkalibacillus*, *Lysinibacillus*/*Sporosarcina*, and *Ruegeria*/*Cribrihabitans* clusters showed resistance and robust growth under metal mixtures. Among all isolates, 11, 32, 81, and 82 (*Bacillus*/*Mesobacillus*/*Cytobacillus* and *Bacillus*/*Peribacillus*/*Rossellomorea*) combined broad multi-metal tolerance with strong biofilm formation, positioning them as candidates for site-specific, nature-based bioremediation of heavy-metal-impacted coastal ecosystems such as Šibenik Bay.

## 1. Introduction

Heavy metals are naturally occurring elements, characterized by high atomic weight and density. Beyond their natural occurrence, they are extensively applied in industry, agriculture, household products, medicine, and technology. Their widespread use has resulted in significant environmental dispersion, raising serious concerns regarding their potential impacts on ecosystems and human health [1]. Although trace amounts of heavy metals including cobalt, copper, chromium, iron, magnesium, manganese, molybdenum, nickel, selenium, and zinc have been reported as essential micronutrients supporting various metabolic processes in living cells, elevated concentrations may become toxic [1,2,3]. Of all heavy metals, mercury, arsenic, cadmium, chromium, and lead are considered to be highly toxic, even at low exposure levels, due to their potential to cause organ damage, and have been classified as known or probable human carcinogens by both the U.S. Environmental Protection Agency and the International Agency for Research on Cancer [1].

Mercury, especially methylmercury, is highly neurotoxic and tends to bioaccumulate and biomagnify in aquatic food webs; arsenic, particularly in its trivalent form (As(III)), is a well-known carcinogen commonly found in contaminated groundwater; cadmium is toxic even at low concentrations and readily bioaccumulates; hexavalent chromium (Cr(VI)) is both highly toxic and carcinogenic with strong environmental mobility; and lead is a persistent metal that bioaccumulates and affects neurological, renal, and developmental systems [4].

Heavy metal pollution in coastal marine zones has emerged as a global concern in recent decades, driven by insufficient regulation and the continuous release of metals, highlighting the urgent need for effective remediation strategies. Coastal areas act as major sinks for heavy metals, with contamination stemming from diverse anthropogenic activities, such as mining and smelting, industrial operations, agricultural and household use of metal-containing products, untreated wastewater discharge, and shipyard activities [5,6,7]. The Mediterranean region is particularly vulnerable, as intensive tourism imposes additional pressure on already stressed coastal ecosystems [8]. While traditional methods for removing heavy metals from contaminated environments, such as chemical precipitation, ion exchange, electrochemical treatment, and adsorption, have been widely used, they often come with significant limitations [9]. These include high operational costs, the generation of secondary pollutants, inefficiency at low metal concentrations, and unsuitability for large-scale or in situ applications. In contrast, microbial bioremediation is increasingly being explored as a sustainable, safe, and cost-effective alternative, including many existing studies on the marine environment [10]. Out of all microorganisms, bacteria have shown great potential as effective agents capable of transforming heavy metals into less or non-toxic forms [11,12,13]. Microbial consortiums, biofilms, and genetically engineered organisms are examples of technological advancements that have increased the efficacy of bioremediation tasks [10,14]. Most microorganisms follow common mechanisms for the removal of heavy metals from environments, including the secretion of extracellular barriers, extracellular and intracellular sequestration, active transport of metal ions, production of microbial metabolites acting as chelating agents, and enzymatic detoxification [11,13,15]. The bioremediation process could also be accomplished in aerobic and anaerobic environments; however, the aerobic environment was found to be faster and more efficient than anaerobic conditions. To date, many metal-resistant bacteria have been identified that are promising for the bioremediation of contaminated marine sediments. *Pseudomonas stutzeri* W228 demonstrated the ability to remove copper and lead [16], while rhamnolipids produced by *Pseudomonas aeruginosa* enhanced the removal of Pb and Hg from intertidal sediments [17]. Furthermore, sulfate-reducing bacterial consortia were reported to immobilize heavy metals in estuarine sediments through in situ biomineralization, reducing their mobility and ecological risk [18].

The bioremediation potential of microorganisms results from their ability to develop adaptive mechanisms for survival in heavy metal-polluted contaminated environments: in particular, the evolution of their resistome and the ability to form biofilms. Biofilm formation is particularly important for bioremediation, as extracellular polymeric substances (EPS) enhance microbial survival under toxic conditions while promoting immobilization, transformation, and removal of metals from the environment [19,20,21,22,23]. In marine ecosystems, microbial communities are additionally challenged by dynamic and stressful conditions, including fluctuating pH, salinity, temperature and sea currents. These drive the evolution of specialized adaptive traits, further reinforcing the potential of marine microorganisms as one of the most promising agents for the bioremediation of contaminants such as heavy metals. Finally, the use of site-specific indigenous bacteria is particularly promising in bioremediation, as these strains have already adapted to local contamination pressures as well as environmental conditions. Consequently, many studies, both in saline and freshwater, showed that indigenous microbes demonstrate the capability to effectively eliminate heavy metals and are better integrated into the native microbial community, reducing ecological disruption and increasing the likelihood of successful, sustainable remediation [10,24,25].

The environmental quality of the sediment in Šibenik Bay, located on the eastern Adriatic coast, has been significantly affected by various anthropogenic activities over the years. As a result, numerous studies and ongoing environmental monitoring programs have consistently reported elevated concentrations of various trace metals in the bay’s sediments [26,27,28,29,30,31,32,33,34]. In particular, high levels of Hg with concentrations of up to 3.53 mg/kg have been previously reported, along with Cd, Zn, Pb, As, and Cu, which were found in ranges from 24 mg/kg (Cu) to 4010 mg/kg (Zn) [26,28]. Although several major sources of pollution—such as the production of electrodes and ferroalloys, ship repair, and the discharge of untreated wastewater—have ceased, no significant decrease in trace metal concentrations in surface sediments has been observed. This indicates a persistent contaminated site where metal concentrations continue to be of ecological concern and pose a significant threat to the bay’s ecosystem. Our own recent investigation [34] revealed very high to extremely high enrichment of surface sediments [35] with various trace metals including Ba, Bi, Cd, Cr, Cu, Hg, Mo, Pb, Sb, Sn and Zn. Cukrov et al. [26] suggested that Hg contamination at some micro-locations is comparable to some of the most heavily polluted regions of the Mediterranean. Polluted environments such as Šibenik Bay thus represent valuable reservoirs of novel, metal-resistant bacteria with the potential to address long-term environmental damage through site-specific remediation strategies.

Previous research on the sediments of Šibenik Bay has largely focused either on the assessment of heavy metal contamination [26,27,28,29,30,31,32,33] or the study of microbial communities [34], but rarely on both aspects together. This study fills this gap by simultaneously evaluating heavy metal levels and tolerance profiles of indigenous bacterial isolates, providing a more holistic view of metal stress-adapted microbial populations. Using traditional culture-based methods, we isolated strains from sediment samples and applied several complementary approaches to evaluate functional traits relevant to heavy metal removal, including tolerance to individual metals and mixtures—conditions that reflect their natural exposure—as well as biofilm-forming ability. To our knowledge, this is one of the first comprehensive studies of indigenous metal-resistant bacteria from the chronically polluted sediments of Šibenik Bay, highlighting the potential of local strains to be promising agents for sustainable, ecosystem-tailored bioremediation.

## 2. Materials and Methods

### 2.1. Study Sites in the Central Adriatic Sea (Croatia), Sediment Sampling, and Chemical Characterization

**Figure 1:**
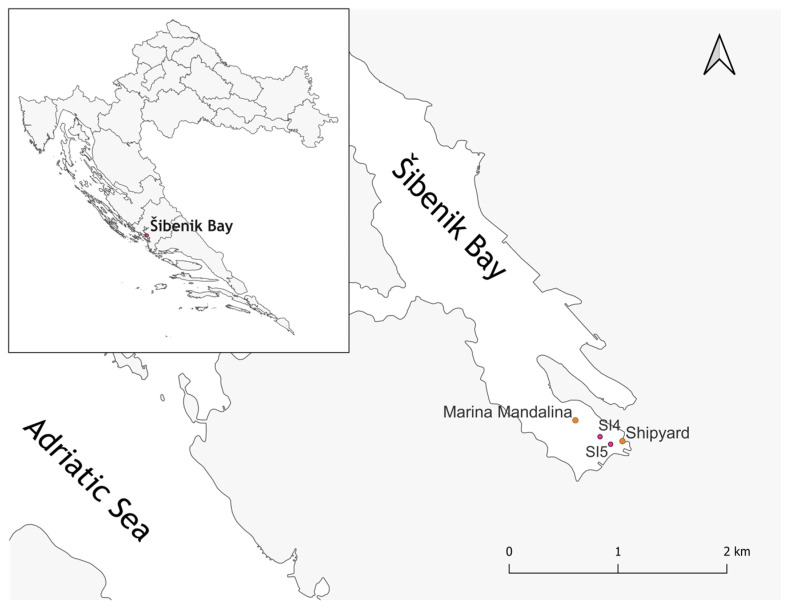
Map of the Šibenik Bay sampling area highlighting specific microsites SI4 and SI5, where sediment samples were collected.

For the isolation of individual bacteria, fresh sediment samples (upper 5 cm sediment layer, sandy silt according to the Shepard classification) were taken from specific microsites in Šibenik Bay: SI4 (43°43′1.077″; 15°54′7.5918″) and SI5 (43°42′58.8636″; 15°54′11.9478″) (Figure 1). The microsites were selected based on the previously observed elevated concentrations of metals in this area [28,29,30,31,34] and following observations that metal accumulation occurs mainly near the source: in this case, the shipyard and marina. Sediment samples were collected from the boat using a Van Veen grab (Hydro-Bios GmbH, Kiel, Germany) and stored immediately at 4 °C until processing (within the first 24 h). Sediment samples were freeze-dried (Freezone 2.5, Labconco Corporation, Kansas City, MO, USA) and digested in a microwave oven (Multiwave 3000, Anton Paar, Graz, Austria) prior to geochemical analysis. The digestion procedure was performed in a two-step procedure: I—5 mL HNO_3_  (65%, pro analysis, Kemika d.d., Zagreb, Croatia)  +  1 mL HCl (37%, VLSI Grade, Rotipuran, Carl Roth GmbH + Co. KG, Karlsruhe, Germany)  +  1 mL HF (47–51%, for trace analysis, Honeywell Research Chemicals (Fluka®), Seelze, Germany); and II—6 mL H_3_BO_3_ (40 g l^−1^, Honeywell Research Chemicals (Fluka®), Seelze, Germany).

The total metal concentrations were determined using an inductively coupled plasma triple quadrupole mass spectrometer (ICP-MS/MS, 8900, Agilent Technologies Inc., Santa Clara, CA, USA). Analytical procedures followed Fiket et al. [36] as previously applied in Ramljak et al. [34]. Measured concentrations were recalculated into enrichment factor (EF) values, a commonly used approach for assessing anthropogenic impact on sediments using the formula EF = E/E_BN_; E_BN_ = f(E_REF_), where E_BN_ indicated background normalization and E_REF_ the reference element: in this case, aluminum (Al) [34,37,38,39].

### 2.2. Isolation and Identification of Bacterial Isolates from Sediment

Bacteria were isolated from freshly collected sediments by inoculating 1 mL of the sediment suspension (prepared by mixing one gram of sediment in 100 mL of artificial seawater) onto Difco™ Marine Agar plates (Becton, Dickinson and Company, Franklin Lakes, NJ, USA). The plates were incubated at 26 °C until colony growth was visible. Individual bacterial colonies, selected according to their morphological appearance, were picked from the plates with a sterile loop and subcultured several times on fresh marine agar plates until pure isolates were obtained. For DNA extraction from the bacterial isolates, the Quick-DNA™ Miniprep Plus Kit (Zymo Research, Irvine, CA, USA) was used according to the manufacturer’s protocol. The DNA concentration was measured with the QUBIT 3.0 fluorometer (Thermo Fisher, Waltham, MA, USA).

To identify the isolates, the 16S rRNA marker gene was amplified using standard primers (27F and 1492R), following the protocol of [40]. The success of the amplification was confirmed by agarose gel electrophoresis. PCR products (~1400 base pairs) were sent for Sanger sequencing to a commercial provider (Macrogen, Amsterdam, The Netherlands). The resulting raw sequences were manually edited using Chromas Lite v2.6.6 (Technelysium, South Brisbane, Australia) and compared against the National Center for Biotechnology Information (NCBI) database using the BLASTn tool (version 2.16.1+). A neighbor-joining phylogenetic tree based on the 16S rRNA gene sequences was constructed using ClustalX (version 2.1.) and MEGA-X (version 10.0.5.) and visualized with the Interactive Tree of Life (iTOL version 7.2.2.) online tool [41]. The tree was built using 1000 bootstrap replicates and a substitution rate of 0.01 per nucleotide position to determine the phylogenetic placement of the isolates and closely related species.

### 2.3. Testing the Resistance of Bacterial Isolates to Individual Metals Using the Disk Diffusion Method

**Figure 2:**
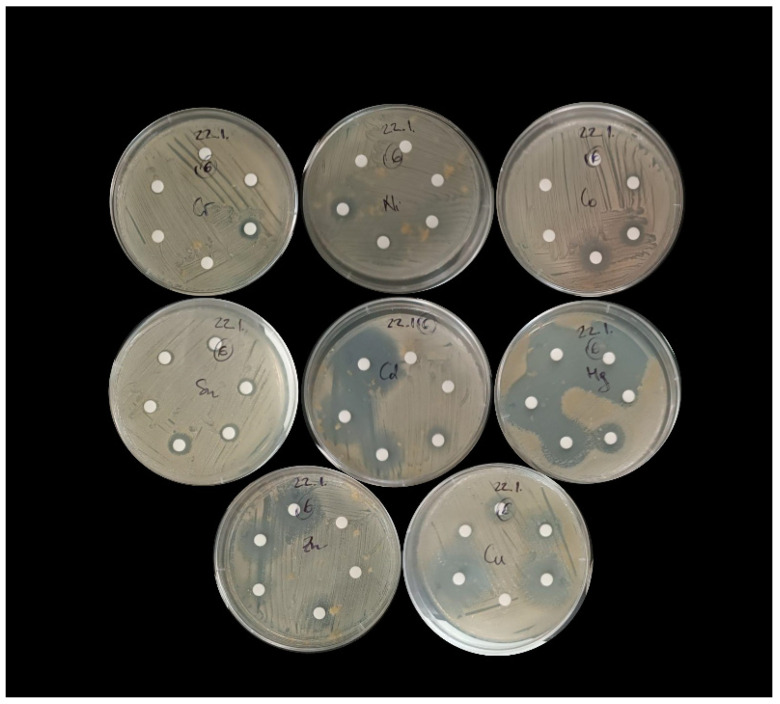
An example of the disk diffusion method used to test bacterial resistance to selected metals, tested at concentrations of up to 5000 mg/L for Zn, Cr, Cd, Hg, and TBT, and up to 10,000 mg/L for Sn, Pb, Cu, Co, and Ni.

When testing the resistance of bacterial isolates to different concentrations of nine individual heavy metals, the following were selected: Cd, Co, Cr, Cu, Hg, Ni, Pb, Sn, and Zn. A list of the compounds used for the experiment and the concentration ranges used can be found in Supplementary (Table S1). The required salt weight was calculated based on the molecular weight of the salt and the atomic weight of the target metal ion. Bacterial resistance to selected metals was determined using the disk diffusion method [42]. In brief, 24 h-old cultures of the individual pure bacterial isolates grown in Difco™ Marine Broth (Becton, Dickinson and Company, Franklin Lakes, NJ, USA) were first diluted (with 0.85% NaCl) to the required concentration of 0.5 McFarland. After a sterile swab was dipped into the bacterial suspension, the entire surface of each Difco™ Marine Agar plate (Becton, Dickinson and Company, Franklin Lakes, NJ, USA) was swabbed. Six empty cellulose disks (6 mm) were placed in a circle on a Petri dish inoculated with bacteria (Figure 2). We applied 10 µL of the prepared individual metal stock solution (Table S1) to each of the disks. The radii of the zones of inhibition were measured after 24 h of incubation of the disks at 26 °C, and the maximum tolerance concentration (MTC) for each isolate was determined from the clear zone around the disk (Figure 2). Bacterial susceptibility was inferred from the absence of growth, while the presence of growth indicated bacterial resistance.

### 2.4. Testing the Resistance of Bacterial Isolates to Metal Mixtures in 96-Well Plates

**Figure 3:**
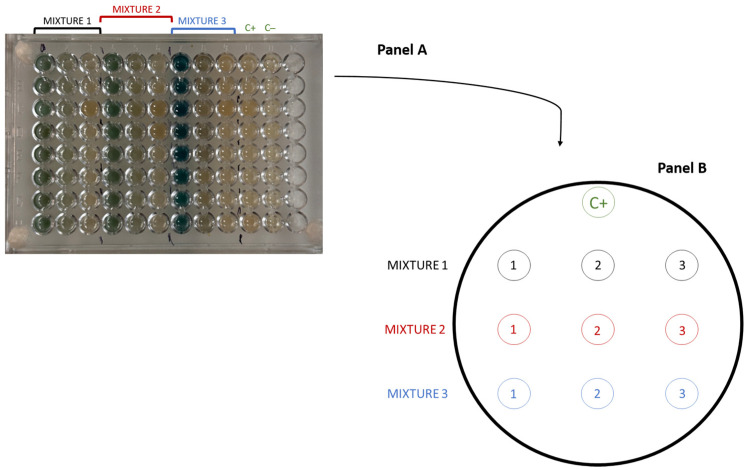
Schematic representation of the experimental design for testing bacterial isolate resistance to metal mixtures using 96-well plates (Panel (**A**), and later on, spot-inoculation onto agar plates (Panel (**B**). C+—positive control; C−—negative control; 1—concentration 1000 mg/L, 2—concentration 100 mg/L, 3—concentration 10 mg/L. Mixture 1—Cd, Co, Cr, Cu, Hg, Ni, Pb, Sn, and Zn; Mixture 2—Cd, Co, Cr, Cu, Ni, Pb, Sn, and Zn; Mixture 3—Cd, Co, Cr, Cu, Ni, and Zn.

The resistance of individual bacterial isolates to heavy metal mixtures was tested in Difco™ Marine Broth (Becton, Dickinson and Company, Franklin Lakes, NJ, USA), using a 96-well microtiter plate method. For each assay, 24 h-old pure cultures grown in the same broth were used. Three different metal mixtures were prepared: (i) Mixture 1, which contained all 9 metals: Cd, Co, Cr, Cu, Hg, Ni, Pb, Sn, and Zn; (ii) Mixture 2, which was identical to Mixture 1 but without Hg; and (iii) Mixture 3, which was identical to Mixture 1 but without Hg, Pb, and Sn. Each metal in the mixtures was tested at three concentrations: 1000 mg/L, 100 mg/L, and 10 mg/L. Each well of a sterile 96-well plate was filled with Marine Broth containing the corresponding metal mixture at the indicated concentration and 280 µL of overnight grown bacterial cultures diluted to an OD of 2 in Difco™ Marine Broth. For each isolate, a positive control (C+) was performed by inoculating the strain into the wells with broth, without added metals, while the negative control (C−) included the addition of broth media only (Figure 3A). The plates were incubated at 26 °C for 24 h. Then, 20 µL from each well was spot-inoculated onto Difco™ Marine Broth Agar (Becton, Dickinson and Company, Franklin Lakes, NJ, USA) for visualization of bacterial growth according to the scheme shown in Figure 3B. Plates were incubated at 26 °C for 24 h or 48 h (slower growth rate strains). The concentration at which no colony growth was observed was designated as the growth-limiting concentration.

### 2.5. Assessment of Biofilm-Forming Ability of Bacterial Isolates

The biofilm-forming ability (BFA) of bacterial isolates was assessed using the Crystal Violet assay, following the method described by O’Toole [43]. Three-day-old bacterial colonies were initially transferred to Difco™ Marine Broth (Becton, Dickinson and Company, Franklin Lakes, NJ, USA) and incubated for an additional 18 h at 25 °C. A 10 μL aliquot of the bacterial suspension was then added to 90 μL of Marine Broth in four round-bottom wells of a polystyrene 96-well plate (Isolab GmbH, Eschau, Germany). Control wells contained 100 μL of sterile Marine Broth. To minimize evaporation and prevent the use of corner wells, the first and last columns of the plate were filled with 100 μL of sterile distilled water. The plates were incubated at 25 °C for 24 h, after which they were washed three times and stained with 0.1% Crystal Violet solution (Merck, Darmstadt, Germany) for 15 min at room temperature (25 ± 2.0 °C). Once air-dried, the stain was dissolved using 125 μL of 96% ethanol (Kemika d.d., Zagreb, Croatia) per well. After a 15 min extraction, 100 μL of the solution was transferred to a new flat-bottom 96-well plate, and absorbance was measured at 588 nm, using a microplate reader (Spectrostar Nano, BMG Labtech, Ortenberg, Germany). The BFA of the separate isolate was represented as an average absorbance at 588 nm of four inoculated wells. The success of isolates in the biofilm formation was assessed by calculating the average BFA of all isolates and comparing the average BFA value with individual BFA values. All isolates that formed more biofilm than average were considered to be good biofilm formers.

## 3. Results

### 3.1. Measured Levels of Metals in Sediment Samples

**Figure 4:**
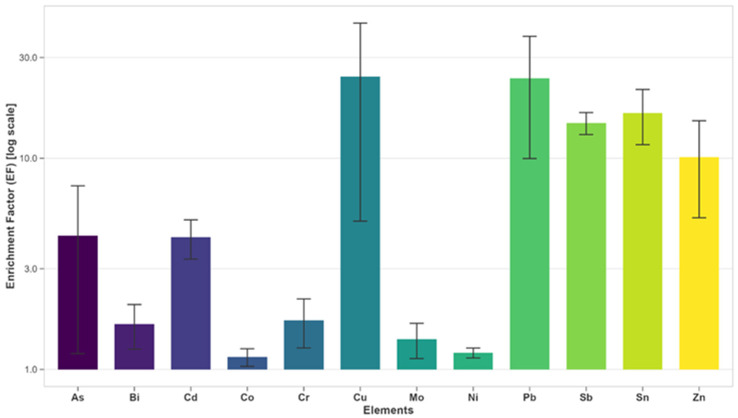
Measured enrichment factors of 12 elements in sediment samples collected from microsites SI4 and SI5 in Šibenik Bay. The bars represent average values for SI4 and SI5, while the asymmetrical error bars reflect the inherent variability in metal concentrations at these sites, due to their varying proximity to point pollution sources.

To assess sediment contamination levels, metal concentration data were used to calculate enrichment factors (Figure 4). Analysis of the metal concentrations in sediment samples used for bacterial isolation revealed the presence of all targeted metal(loid)s at varying levels. Based on enrichment classification [35], moderate enrichment, which is indicative of moderate pollution, was observed for As, Cd, and Cr. In contrast, Cu, Pb, Sb, Sn, and Zn showed significant enrichment, reflecting strong pollution signals. At site SI5, Cu and Pb reached particularly high concentrations, corresponding to very high enrichment levels and indicating severe contamination (Table S2). Total element concentrations of metal(loid)s used for enrichment factor calculations are shown in Table S3.

### 3.2. Phylogenetic Affiliation of Bacterial Isolates

**Figure 5:**
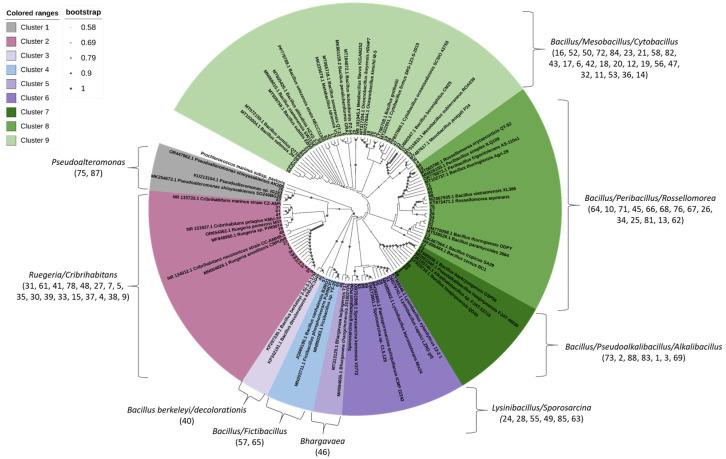
Neighbor-joining tree, based on 16S rRNA gene sequences, showing the phylogenetic positions of bacterial strains isolated from polluted sediment sample from Šibenik Bay.

A total of 74 pure bacterial isolates were successfully recovered and purified from contaminated sediments from Šibenik Bay by cultivation on marine agar plates. The 16S rRNA gene sequences of these isolates were used to construct a phylogenetic tree (Figure 5). Phylogenetic analysis revealed sequence similarities of 98% to 100% to reference sequences in the NCBI GenBank database, including members of the Phyla *Proteobacteria* (*α*- and *γ-Proteobacteria*) and *Firmicutes*. Owing to the high conservation of the 16S rRNA gene among closely related taxa, genus-level classification was not possible for all isolates. As a result, some phylogenetic clusters comprised multiple genera with highly similar or overlapping sequence identities. Based on neighbor-joining tree analysis, the isolates were grouped into nine distinct taxonomic clusters: *Pseudoalteromonas* cluster 1 (2 isolates; 2.7%), *Ruegeria*/*Cribrihabitans* cluster 2 (17 isolates; 23.0%), *Bacillus berkeleyi*/*decolorationis* cluster 3 (1 isolate; 1.4%), *Bacillus*/*Fictibacillus* cluster 4 (2 isolates; 2.7%), *Bhargavaea* cluster 5 (1 isolate; 1.4%), *Lysinibacillus*/*Sporosarcina* cluster 6 (6 isolates; 8.1%), *Bacillus*/*Pseudoalkalibacillus*/*Alkalibacillus* cluster 7 (7 isolates; 9.5%), *Bacillus*/*Peribacillus*/*Rossellomorea* cluster 8 (14 isolates; 18.9%), and *Bacillus*/*Mesobacillus*/*Cytobacillus* cluster 9 (24 isolates; 32.4%). The majority of bacterial isolates belonged to three phylogenetic clusters: Cluster 9 (*Bacillus*/*Mesobacillus*/*Cytobacillus*), Cluster 2 (*Ruegeria*/*Cribrihabitans*) and Cluster 8 (*Bacillus*/*Peribacillus*/*Rossellomorea*), which together accounted for over 74% of all recovered isolates, indicating their prevalence in the polluted sediment environment or their feasibility to be isolated in the laboratory. The 16S rRNA sequences of pure bacterial isolates have been deposited in the NCBI GenBank database under accession numbers PV643243-PV643316.

The tree was constructed using the K2 + G model (Kimura 2 + Gamma distribution). The analysis was performed based on 1000 replications.

### 3.3. Tolerance of Sediment Bacterial Isolates to Individual Metals

Following exposure of each pure bacterial isolate to nine tested metals using the disk diffusion method, minimal MTC values, defined as the concentration completely inhibiting bacterial growth, were determined for each isolate. The complete MTC dataset for each of the individually tested isolates is provided in Supplementary Table S4, while percentage distribution of bacterial isolates exhibiting complete growth inhibition at the defined concentration is summarized in Table 1 and Table 2. For clarity, the tested metals were categorized into two groups based on their toxicity and the concentration ranges applied: metals with higher toxicity (tested up to 5000 mg/L, Table 1), and metals with lower toxicity (tested up to 10,000 mg/L, Table 2).

Among all the metals tested, Hg exhibited the highest toxicity, with over 90% of isolates inhibited at a concentration of just 50 mg/L, and only 2 (isolate 21-Cluster 9 and 68-Cluster 8) out of 74 isolates remained sensitive at 500 mg/L. For Sn, the MTC threshold at which more than 50% of the community members’ growth was inhibited ranged between 100 and 500 mg/L. Sn-resistant strains (inhibition at 10,000 mg/L or more) included isolates 3 (Cluster 7), 13, 68, 76, 81 (Cluster 8), 16, 19, 21, 36, 47 and 82 (cluster 9). In the case of Zn, Cr, and Cd, concentrations between 500 and 1000 mg/L were required to inhibit the growth of the majority of community isolates. The 500 mg/L MTC threshold was most frequently observed for Zn, where it inhibited more than 50% of isolates, and for Cd, where it inhibited over 70% of isolates. Highly Zn-resistant strains included isolates 5, 53, 67 and 81 (Clusters 2, 9 and 8), and Cd-resistant included 13 and 36 (Clusters 8 and 9), 25 (both metals, Cluster 8). Cr seems to display a bimodal resistance pattern—beside these 30% of isolates inhibited at 500 mg/L, another 30% showed high resistance, with no growth inhibition even at the maximum tested concentration of 5000 mg/L (23 isolates from different Clusters). Bacteria demonstrated lower sensitivity to Co and Ni, with growth inhibition observed only at high concentrations of 5000 mg/L or 10,000 mg/L. Pb showed the lowest toxicity, with more than 75% of isolates exhibiting resistance, even at concentrations exceeding 10,000 mg/L. In addition to these overall resistance trends, distinct patterns were observed for Cu and Cr. For Cu, approximately 20% of different isolates showed resistance at each of the four tested concentrations—500, 1000, 2500, and 5000 mg/L—indicating a broad and evenly distributed resistance response across the tested range.

Based on the MTC values determined for individual bacterial isolates (Table S4), several strains were selected for their broad spectrum and high resistance to multiple metals. These isolates demonstrated resistance to a majority of the nine tested metals, making them strong candidates for further investigation into metal-resistance mechanisms. Notably, isolate 25 (resistant to Pb, Zn, Cr, Cd, Cu, Co, and Ni), isolate 81 (resistant to Sn, Pb, Zn, Cr, Cu, Co, and Ni), and isolate 13 (resistant to Cr, Cd, Sn, Pb, Co, and Ni), all from cluster 8, exhibited the highest resistance profiles. Additionally, isolate 36 (resistant to Sn, Pb, Cr, Co, and Ni) and isolate 47 (resistant to Sn, Pb, Cr, Cu, Co, and Ni) from cluster 9 also showed notable multi-metal resistance.

### 3.4. Tolerance of Sediment Bacterial Isolates to Heavy Metal Mixtures

**Figure 6:**
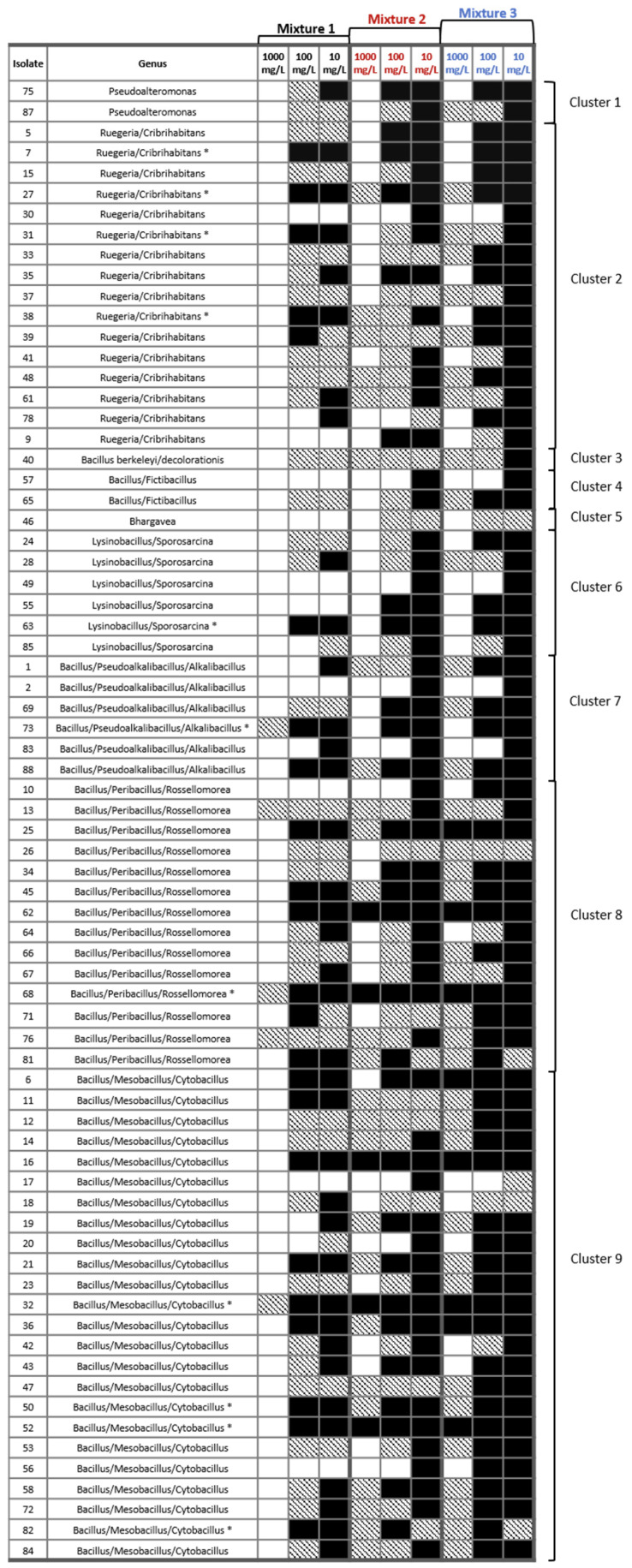
Impact of three multi-metal mixtures (Mixtures 1–3) at different concentrations on bacterial isolates. Black = no change compared to control, dashed = altered colony color/shape, white = growth inhibition. The highest tolerant isolates are marked with an asterisk (*).

The growth inhibition of individual isolates exposed to mixtures of different metals were tested in a liquid culture experiment (Figure 6). Overall, the success of isolate growth followed a consistent trend across metal mixtures: Mixture 3 (excluding Hg, Pb, and Sn) > Mixture 2 (excluding only Hg) > Mixture 1 (containing all nine metals), and concentration 10 mg/L > 100 mg/L > 1000 mg/L. Additionally, for many isolates, growth was observed, but accompanied by noticeable changes in colony morphology or color. Based on observed growth patterns, several isolates were classified as either sensitive or resistant to metal exposure. Sensitive isolates included 10 strains: 30 and 78 (Cluster 2), 57 (Cluster 4), 46 (Cluster 5), 49 and 55 (Cluster 6), 2 and 85 (Cluster 7), 10 (Cluster 8), and 17 (Cluster 9). In contrast, a significantly larger group of highly resistant isolates was identified, comprising 24 strains: 6, 11, 16, 32, 36, 47, 50, 52, 81, and 82 (Cluster 9); 13, 25, 45, 62, 68, and 76 (Cluster 8); 69 and 73 (Cluster 7); 63 (Cluster 6); and 7, 27, 31, 38, and 75 (Cluster 2). Additionally, a subset of 11 isolates showed robust growth, even in the presence of all nine metals: 82, 52, 50, and 32 (Cluster 9); 68 (Cluster 8); 73 (Cluster 7); 63 (Cluster 6); and 38, 31, 27, and 7 (Cluster 2).

### 3.5. Biofilm-Forming Ability of Bacterial Isolates

**Figure 7:**
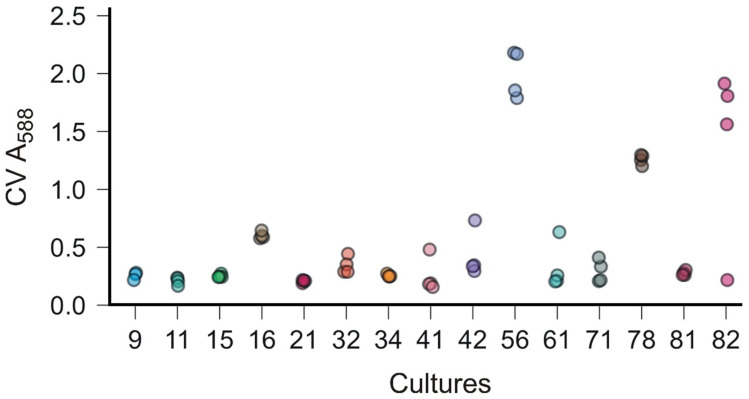
Biofilm-forming ability of bacterial isolates measured by microtiter plate assay (Crystal Violet -CV) at 588 nm. Dots (quadruplicate measurements) represent isolates with above-average biofilm formation (mean absorbance > 0.2). Isolate numbers are shown on the *x*-axis and the colors represent quadruplicates of each isolate.

The biofilm-forming ability trait was tested in all isolates: 15 of them had an above-average BFA value, while 11 isolates did not form a biofilm in the present microtiter test. The average absorbance value of all isolates at 588 nm was 0.2, while the highest absorbance of the above-average biofilm formers was 2.18. Out of 15 good biofilm formers, seven isolates belonged to the *Bacillus*/*Mesobacillus*/*Cytobacillus* Cluster 9 (isolates 11, 16, 21, 32, 42, 56, and 82), the next five to the *Ruegeria*/*Cribrihabitans* Cluster 2 (isolates 9, 15, 41, 61, and 78) and the last three to the *Bacillus*/*Peribacillus*/*Rossellomorea* Cluster 8 (isolates 34, 71, and 81) (Figure 7).

## 4. Discussion

Šibenik Bay is a complex marine environment influenced by both natural processes and long-term anthropogenic activities, with elevated concentrations of heavy metals historically measured in its sediments and water. Such conditions create strong selection pressures that shape the composition and functional traits of the resident microbial communities. To investigate the adaptive responses of sediment-associated bacteria to long-term metal contamination, we isolated and evaluated their resistance to multiple metals. These findings provide insights into microbial adaptation and co-selection processes, while the recognition of strains with distinct multi-metal resistance and the associated functional BFA trait highlights their potential for environmentally sustainable bioremediation strategies in this area.

Bacterial isolates from the metal-polluted sediments of Šibenik Bay displayed varying degrees of tolerance (MTC) to the nine tested heavy metals (Cd, Co, Cr, Cu, Hg, Ni, Pb, Sn, Zn). Overall, metal toxicity followed the trend Hg > Sn > Zn/Cd/Cr > Co/Ni > Pb, with Cu showing a more evenly distributed resistance profile across the tested range (500–5000 mg/L), without a clear single threshold for the majority. As expected, Hg proved to be the most toxic, with our isolates showing the lowest tolerance (inhibited at only 50 mg/L), which is consistent with its well-documented high reactivity, mobility, and bioaccumulative behavior that makes it one of the most concerning contaminants in aquatic environments [44,45,46]. Low metal tolerance was also observed for Zn, Cr, Cd, and Sn—Sn limited growth in concentrations between 100 and 500 mg/L, while Zn, Cd, and Cr generally limited growth at 500–1000 mg/L, although Cr displayed a bimodal pattern, with some strains remaining resistant even at 5000 mg/L. In contrast, strong adaptation was evident for Co, Ni, and particularly Pb, with inhibition occurring only at 5000–10,000 mg/L, and beyond 10,000 mg/L, respectively. The presence of resistance traits against all tested metals, including multi-metal resistance to as many as seven heavy metals in some isolates, reflects the strong and persistent selective pressures shaping microbial communities in Šibenik Bay. These findings are in line with previous reports showing that bacterial communities in metal-polluted environments often evolve resistance mechanisms to certain pollutants while remaining susceptible to others [47,48], with potential origins linked to genetic adaptations such as metal efflux systems, enzymatic detoxification, or biofilm formation [21], underscoring the need for further investigation of these traits in our isolates. The observed high metal-resistance levels, particularly against toxic metals, suggest chronic exposure to these contaminants. However, since no standardized metal tolerance breakpoints (MTC) exist that are comparable to MIC values for antibiotics, the values obtained in this study are context-dependent and cannot be directly compared across studies without standardized methods. Establishing such standards would greatly improve comparability across studies and advance our understanding of microbial meta-resistance in diverse environments.

Interestingly, the observed resistance profiles did not fully align with sediment chemistry, as recent measurements [34] showed high enrichment factors not only for Pb but also for Cd, Sn, Zn, and Cu, whereas Co and Ni levels remained low. This decoupling between sediment chemistry and bacterial resistance profiles suggests that factors beyond bulk metal concentrations, such as bioavailability, metal speciation, or historical exposure patterns play key roles in shaping benthic community microbial tolerance. Even though sediments in Šibenik Bay show high enrichment factors for several metals, not all of these are equally bioavailable to microbes. Metals bound to sulfides or organic matter and within residual fraction may be less accessible and thus exert weaker selective pressure than their measured total concentrations would suggest. Conversely, repeated historical pulses of particular metals (e.g., Pb, Ni, Co) may have provided consistent selective pressure, favoring stable resistance traits, regardless of current abundance. In addition, resistance can spread through mobile genetic elements, meaning the presence of one dominant metal may co-select for resistance to others, further complicating the link between sediment chemistry and microbial profiles.

The *Bacillus* species dominated the sediment samples, with 48 isolates distributed across five closely related clusters, highlighting this genus as the principal microbial group thriving in the heavy metal-polluted environment of Šibenik Bay. Isolates 25, 81, and 13 from *Bacillus*/*Peribacillus*/*Rossellomorea* cluster, together with isolates 36 and 47 from *Bacillus*/*Mesobacillus*/*Cytobacillus cluster* exhibited the strongest individual metal resistance profiles, highlighting *Bacillus*-related lineages as key reservoirs of multi-metal tolerance. Genus *Rossellomorea* has recently been isolated from sediment samples in China, exhibiting resistance to both the antibiotic lincomycin and copper [49]. Additionally, a similar strain, *Rossellomorea arthrocnemi*, has been used in the phytoremediation of heavy metal-polluted soils [50]. *Mesobacillus jeotgali*, isolated from coastal sediments, has been tested as a biosorbent, confirming its efficacy for Cd and Zn removal from coastal environments [51]. Furthermore, *Cytobacillus pseudoceanisediminis*, closely related to *Cytobacillus oceanisediminis*, was isolated from a deep subsurface saline spring and exhibited tolerance to high concentrations of Cd, Cu and Pb [52]. Concerning *Bacillus* species, they are frequently associated with heavy metal-contaminated environments and are well known for their multi-metal resistance [53,54]. Several studies have documented their metal resistance profiles: Zampieri et al. [54] identified various *Bacillus* strains with differing resistance levels to Cd, Cr, Cu, and Zn; Domingues et al. [53] reported the dominance of *Bacillus* (Pb, Zn) and *Alcaligenes* (Pb, As, Cd, Zn); and Kalkan et al. [13] described *Bacillus* strains capable of coping with 16 different heavy metals. Although *Bacillus* species are often isolated from contaminated environments and exhibit high tolerance and diverse detoxification mechanisms, the dominance of indigenous strains in Šibenik Bay suggests that their long-term adaptation to local metal pollution may make them particularly effective candidates for site-specific applications tailored to the conditions of Šibenik Bay.

Beyond the well-known *Bacillus* group, our study revealed several less-explored genera—*Ruegeria*/*Cribrihabitans, Bhargavaea, Pseudoalteromonas*, and *Lysinibacillus*/*Sporosarcina*—with notable resistance traits, underscoring their potential as novel candidates for heavy metal bioremediation in Šibenik Bay. Even though *Ruegeria* species are prevalent in marine environments and are known for their adaptability to various stressors, currently there are no similar studies that directly associate Ruegeria/*Cribrihabitans* species with mechanisms of heavy metal resistance. However, their ability to form biofilms and produce exopolysaccharides enhances their survival in heavy-metal-contaminated habitats, making them suitable candidates for bioremediation applications. Similarly, the bioremediation potential of *Bhargavaea beijingensis* has only been recognized in two recent studies [55,56]. These studies demonstrated the substantial resistance of *Bhargavaea* to multiple heavy metals and active hyperaccumulation, particularly of Hg. Additionally, genomic analysis in these studies revealed a novel repUS12-type plasmid carrying the *ermT* and *tet(L)* genes. This provided antibiotic resistance, suggesting an adaptive advantage under combined heavy metal and antimicrobial pressures, and enhancing the bacterium’s survival and functional performance in contaminated environments. Finally, several studies identified *Lysinibacillus* and *Sporosarcina* as species exhibiting diverse mechanisms to cope with heavy metal stress, including bioaccumulation, enzymatic detoxification, and biomineralization [57,58,59]. The genome of *L. sphaericus* OT4b.31 contains multiple resistance operons (e.g., *nik*, *ars*, *czc*, *cop*, *chr*, *czr*, *cad*), providing resistance to metals such as Ni, As, Cd, Cr, and Cu. *Sporosarcina* sp. strain G3 shown ability to bioaccumulate Cd, Co, and Zn, and produces MerA and chromate reductase enzymes for mercury and chromate detoxification. In addition, *S. pasteurii* employs microbially induced calcite precipitation (MICP) to immobilize Cd, Cu, and Pb as carbonates, reducing their bioavailability. These traits position all these species as promising candidates for bioremediation applications targeting heavy metal pollution. The discovery of less-explored genera with strong resistance traits expands the pool of potential candidates, pointing to previously overlooked microbial resources that may be equally valuable for future bioremediation strategies.

In our study, we extended tolerance testing to mixtures of metals, since bacteria in natural environments such as Šibenik Bay are simultaneously exposed to multiple contaminants. This approach is particularly important here, because the bay’s sediments contain diverse and historically elevated levels of heavy metals, meaning that microbial survival depends on coping with combined, rather than single-metal, stresses. Ultimately, the ability to tolerate complex multi-metal mixtures is a key feature in identifying strains with strong potential for effective bioremediation in Šibenik Bay and similar contaminated environments. The observed growth patterns indicate that bacterial resistance is both metal-specific and concentration-dependent. Isolate growth followed a consistent pattern across the metal mixtures, with the highest growth in mixture lacking Hg, Pb, and Sn; intermediate growth in mixture lacking only Hg; and the lowest growth in mixture containing all nine metals. The mixture excluding Hg, Pb, and Sn exerted the weakest inhibitory effect on bacterial growth, emphasizing the pronounced toxicity of these three metals when present within complex environmental mixtures. The toxicity of the Hg, Pb, and Sn compounds to bacterial communities is well documented in many studies [60,61]. Additionally, observed changes in colony morphology and pigmentation under these metals suggest activation of physiological defense mechanisms, such as efflux pump systems and enzymatic transformation, consistent with mechanisms reported in other metal-resistant bacteria [12,21,62]. Importantly, 11 isolates—including *Bacillus*/*Mesobacillus*/*Cytobacillus* strains 82, 52, 50, and 32; *Bacillus*/*Peribacillus*/*Rossellomore* strain 68; *Bacillus*/*Pseudoalkalibacillus*/*Alkalibacillus* strain 73; *Lysinibacillus*/*Sporosarcina* strain 63; and *Ruegeria*/*Cribrihabitans* strains 38, 31, 27, and 7—exhibited resistance and robust growth across all three mixtures. This finding indicates exceptional multi-metal resistance and underscores the strong, widely distributed resistance present across several phylogenetic clusters. Such isolates are promising candidates for bioremediation of marine environments where multiple metals co-exist, as often found in long-term polluted coastal areas [12]. A notable aspect of this study is the application of a novel experimental approach, designed to simulate environmentally realistic multi-metal exposures, which has not been previously validated in the literature. This approach provides insights into how bacterial communities respond to metal mixtures, rather than single-metal exposures, and underscores the importance of investigating multi-metal interactions in future bioremediation studies [63]. Future work should focus on characterizing the genetic mechanisms underlying the observed resistance, as well as evaluating the performance of these isolates in simulated or field-scale sediment remediation experiments.

One of the microbial traits that could contribute to metal resistance is biofilm formation, through the protective and sorptive properties of the extracellular polymeric matrix, which enhances microbial survival and promotes metal immobilization under toxic heavy metal conditions [19,21]. We also evaluated this trait in all isolates to identify potentially effective strains for bioremediation of marine environments. Only 15 isolates (20%) demonstrated an increased biofilm-forming ability, including *Bacillus*/*Mesobacillus*/*Cytobacillus* isolates 11, 16, 21, 32, 42, 56, and 82; *Ruegeria*/*Cribrihabitans* isolates 9, 15, 41, 61, and 78; and *Bacillus*/*Peribacillus*/*Rossellomorea* isolates 34, 71, and 81. Biofilm formation in marine bacteria is particularly relevant for bioremediation, because biofilms provide structural stability, increase surface adhesion to sediments and particulates, and facilitate cooperative metabolic interactions that enhance pollutant degradation and metal sequestration. This trait, therefore, not only provides resistance to heavy metal stress but also enables persistence in dynamic marine environments, where fluctuating salinity, nutrient limitation, and hydrodynamic forces can otherwise reduce microbial activity. Supporting this, Sharma et al. [20] demonstrated that EPS-enclosed biofilms outperform planktonic cells in continuous bioremediation systems, while a comprehensive review further highlighted that biofilm development reduces metal bioavailability and promotes communal tolerance mechanisms. Likewise, early work by Chien et al. [23] and Jasu [22] confirmed that EPS production and microbial consortia within biofilms substantially contribute to heavy metal resistance and removal efficiency.

When metal-resistance profiles were considered alongside biofilm-forming ability (BFA), only four isolates—*Bacillus*/*Mesobacillus*/*Cytobacillus* 32 and 11, *Bacillus*/*Peribacillus*/*Rossellomorea* 81, and *Bacillus*/*Mesobacillus*/*Cytobacillus* 82—emerged as the most promising bioremediation agents. These strains distinguished themselves by coupling broad-spectrum metal resistance with robust biofilm formation: a combination not observed in the majority of other isolates. This dual capacity provides a distinct ecological advantage: biofilm production creates a protective barrier against toxic exposure, while cellular mechanisms such as metal efflux and enzymatic detoxification further strengthen survival under high metal loads. Together, these traits highlight the exceptional adaptive potential of these four lineages in metal-polluted environments. Importantly, their unique profile positions them as prime candidates for nature-based, site-specific bioremediation, offering a sustainable approach to reducing heavy metal pollution and supporting the restoration of coastal sediment health. As Šibenik Bay is part of a marine protected area (MPA) within the Natura 2000 network in Croatia, designated under the EU Habitats Directive (92/43/EEC), but remains under considerable ecological pressure from metal contamination, the implementation of urgent restoration measures is essential. Harnessing these bacterial strains could therefore represent a practical, nature-based solution to complement conservation policies and safeguard the ecological integrity of this vulnerable habitat.

## 5. Conclusions

This study, connecting long-term observed metal pollution with indigenous benthic communities for the first time, confirmed that sediment bacterial communities within the Šibenik Bay have developed multiple metal resistances to withstand heavy metal stress. Bacteria are able to tolerate both single and multiple metals, with more than 20 isolates demonstrating high resistance levels to Cr, Co, Ni, and Pb. Their ability to tolerate multi-metal exposure further highlights their ecological resilience and strengthens their suitability for practical bioremediation applications. Four isolates—11, 32, 82 (*Bacillus*/*Mesobacillus*/*Cytobacillus*), and 81 (*Bacillus*/*Peribacillus*/*Rossellomorea*)—showed a combination of multi-metal tolerance and high biofilm formation capacity. These traits make them promising nature-based agents for site-specific remediation of the historically contaminated Šibenik Bay. Site-specific indigenous bacteria could demonstrate superior adaptability and functional capacity in situ, and thus, deserve increased focus in future bioremediation research. At the same time, however, such high resistance not only reflects the adaptive responses of microbial communities but also indicates potential disruptions in ecological functions in this coastal zone ecosystem, including nutrient cycling and organic matter.

## Figures and Tables

**Table 1:** Percentage distribution of bacterial isolates exhibiting complete growth inhibition at defined concentrations of Zn, Cr, Cd, and Hg, as determined by the disk diffusion method. Maximum tolerance concentration (MTC) represents the lowest concentration at which no visible growth was observed. Concentrations above 5000 mg/L are noted as “>5000 mg/L”.

	Distribution (%) of Bacterial Isolates Based on MTC Values						
Metal	50 mg/L	100 mg/L	500 mg/L	1000 mg/L	2500 mg/L	5000 mg/L	>5000 mg/L
Zn	0	1.3%	52.7% *	**23.0%**	17.6%	1.4%	5.4%
Cr	0	0	**29.7%**	**18.9%**	10.8%	9.5%	**31.1%**
Cd	5.4%	4.1%	**70.3%**	**14.9%**	1.4%	2.7%	1.4%
Hg	**90.5%**	6.8%	2.7%	0	0	0	0

* Values in bold indicate the concentration at which the majority of isolates reached their MTC.

**Table 2:** Percentage distribution of bacterial isolates exhibiting complete growth inhibition at defined concentrations of Sn, Pb, Cu, Co and Ni, as determined by the disk diffusion method. Maximum tolerance concentration (MTC) represents the lowest concentration at which no visible growth was observed. Concentrations above 10,000 mg/L are noted as “>10,000 mg/L.”.

	Distribution (%) of Bacterial Isolates Based on MTC Values						
Metal	100 mg/L	500 mg/L	1000 mg/L	2500 mg/L	5000 mg/L	10,000 mg/L	>10,000 mg/L
Sn	**25.7%**	**28.4%** *	12.2%	10.8%	8.1%	5.4%	9.5%
Pb **	1.4%	0	1.4%	5.5%	8.2%	8.2%	**75.3%**
Cu	1.4%	**27.0%**	16.2%	24.3%	21.6%	5.4%	4.1%
Co	0	0	1.4%	18.9%	**37.8%**	27%	14.9%
Ni	0	0	1.4%	9.5%	**33.8%**	**35.1%**	20.3%

* Values in bold indicate the concentration at which the majority of isolates reached their MTC. ** A total of 73 isolates were tested for Pb resistance.

## Data Availability

The original contributions presented in this study are included in the article/Supplementary Materials. Further inquiries can be directed to the corresponding author.

## Supplementary Materials

The following supporting information can be downloaded at: https://www.mdpi.com/article/10.3390/microorganisms13102326/s1. Table S1: List of metals and concentrations used for the experiments. Table S2: Numerical data showing concentrations of metals shown as enrichment factors (EF) measured in SI4 and SI5 surface sediment samples collected in Šibenik Bay. Colored text indicates the EFs obtained from the measurements in the samples. Table S3: Total element concentrations of metal(loid)s measured in SI4 and SI5 surface sediment samples collected in Šibenik Bay. The concentrations were given in ug g−1. Table S4: Maximal tolerance concentrations (MTCs) for all tested bacterial isolates (n = 74 isolates) and each contaminant. The table is separated based on the ranges of concentrations used, up to 5000 mg/L for Zn, Cr, Cd, Hg and TBT, and up to 10,000 mg/L for Sn, Pb, Cu, Co, Ni.
