# Supplementary material for: Resisting the Toxic Tide: Multi-Metal Resistance of Bacteria Originating from Contaminated Šibenik Bay Sediments
Source: Microorganisms. 2025 Oct 8;13(10):2326. doi: 10.3390/microorganisms13102326 (PMC12566528; doi:10.3390/microorganisms13102326)
Supplement: Supplementary file 1 [file microorganisms-13-02326-s001.zip › microorganisms-3886755-supplementary.pdf]

Supplementary Materials

Table S1. List of metals and concentrations used for the experiments.

Metals			Producer	Purity	Concentration used (μg/L)
zinc sulfate monohydrate	**Zn**	$ZnSO_4$ x H2O	Thermo Fisher Scientific, Waltham, MA, USA	99%	50-5,000
cadmium chloride	**Cd**	$CdCl_2$	Sigma-Aldrich, St. Louis, MO, USA	technical grade	50-5,000
potassium dichromate	**Cr**	$K_2Cr_2O_7$	Sigma-Aldrich, St. Louis, MO, USA	99%	50-5,000
mercury (II) chloride	**Hg**	$HgCl_2$	Sigma-Aldrich, St. Louis, MO, USA	99.50%	50-5,000
nickel (II) chloride	**Ni**	$NiCl_2$	Thermo Fisher Scientific, Waltham, MA, USA	98%	100-10,000
tin (II) chloride dihydrate	**Sn**	$SnCl_2 \cdot 2H2O$	Sigma-Aldrich, St. Louis, MO, USA	98%	100-10,000
copper (II) sulfate	**Cu**	$CuSO_4$	Sigma-Aldrich, St. Louis, MO, USA	99%	100-10,000
lead (II) nitrate	**Pb**	$Pb(NO_3)_2$	Thermo Fisher Scientific, Waltham, MA, USA	99%	100-10,000
cobalt (II) chloride	**Co**	$CoCl_2$	Sigma-Aldrich, St. Louis, MO, USA	98%	100-10,000

Table S2. Numerical data showing concentrations of metals shown as enrichment factors (EF) measured in SI4 and SI5 surface sediment samples collected in Šibenik Bay. Colored text indicates the EFs obtained from the measurements in the samples.

	EF As	EF Bi	**EF Cd**	**EF Co**	**EF Cr**	**EF Cu**	EF Mo	**EF Ni**	**EF Pb**	EF Sb	**EF Sn**	**EF Zn**
**SI4**	2.10	1.92	4.85	1.07	1.40	10.7	1.58	1.15	14.1	13.5	13.0	6.67
**SI5**	6.50	1.36	3.59	1.22	2.03	38.0	1.20	1.24	33.7	16.0	19.8	13.61

EF Value	Enrichment level*
<2	Deficiency to minimal enrichment
2-5	Moderate enrichment
5-20	Significant enrichment
20-40	Very high enrichment
>40	Extremely high enrichment

* Sutherland, R.A. Bed Sediment-Associated Trace Metals in an Urban Stream, Oahu, Hawaii. Environmental Geology 2000, 39, 611–627.

Table S3. Total element concentrations of metal (oid)s measured in SI4 and SI5 surface sediment samples collected in Šibenik Bay. The concentrations were given in ug g^{-1}.

	As	Bi	Cd	Co	Cr	Cu	Mo	Ni	Pb	Sb	Sn	Zn
SI4	27.2	1.92	0.64	7.43	90.1	124.4	1.58	42.4	218.5	5.43	21.6	338.0
SI5	69.3	1.36	0.36	4.43	68.2	230.5	1.20	23.9	273.4	3.35	17.1	359.7

Table S4. Maximal tolerance concentrations (MTCs) for all tested bacterial isolates (n = 74 isolates) and each contaminant. The table is separated based on the ranges of concentrations used, up to 5,000 mg/L for Zn, Cr, Cd, Hg and TBT, and up to 10,000 mg/L for Sn, Pb, Cu, Co, Ni.

			**Metals tested for concentration up to 5,000 ug/L**				**Metals tested for concentration up to 10,000 ug/L**				
Isolate no.	Isolate name	Cluster number and name	**Zn**	**Cr**	**Cd**	**Hg**	**Sn**	**Pb**	**Cu**	**Co**	**Ni**
**1**	**1**	**7** **Bacillus/ Pseudoalkalibacillus/ Alkalibacillus**	100	2,500	500	50	500	2,500	2,500	5,000	10,000
**2**	**2**	**7** **Bacillus/ Pseudoalkalibacillus/ Alkalibacillus**	1,000	> 5,000	500	50	1,000	5,000	1,000	10,000	10,000
**3**	**3**	**7** **Bacillus/ Pseudoalkalibacillus/ Alkalibacillus**	2,500	500	500	50	10,000	2,500	2,500	2,500	5,000
**4**	**4**	**2** **Ruegeria/Cribrihabitans**	2,500	500	500	50	500	2,500	1,000	2,500	10,000
**5**	**5**	**2** **Ruegeria/Cribrihabitans**	> 5,000	> 5,000	500	50	500	5,000	2,500	> 10,000	10,000
**6**	**6**	**9** **Bacillus/Mesobacillus/Cytobacillus**	500	> 5,000	500	50	5,000	10,000	5,000	10,000	>10,000
**7**	**7**	**2** **Ruegeria/Cribrihabitans**	500	> 5,000	500	50	1,000	5,000	1,000	10,000	10,000
**8**	**9**	**3** **Bacillus berkeleyi/decolorationis**	1,000	> 5,000	500	50	500	2,500	2,500	10,000	> 10,000
**9**	**10**	**8** **Bacillus/Peribacillus/Rossellomorea**	500	2,500	500	50	100	5,000	1,000	10,000	10,000
**10**	**11**	**9** **Bacillus/Mesobacillus/Cytobacillus**	500	5,000	100	50	500	> 10,000	2,500	1,000	>10,000

**11**	**12**	**9**	**Bacillus/Mesobacillus/Cytobacillus**	500	1,000	1,000	50	5,000	> 10,000	2,500	2,500	2,500
**12**	**13**	**8**	**Bacillus/Peribacillus/Rossellomorea**	2,500	> 5,000	5,000	50	> 10,000	> 10,000	5,000	> 10,000	>10,000
**13**	**14**	**9**	**Bacillus/Mesobacillus/Cytobacillus**	500	5,000	1,000	50	1,000	> 10,000	2,500	5,000	2,500
**14**	**15**	**2**	**Ruegeria/Cribrihabitans**	1,000	1,000	500	50	500	10,000	2,500	10,000	10,000
**15**	**16**	**9**	**Bacillus/Mesobacillus/Cytobacillus**	500	> 5,000	500	100	10,000	> 10,000	2,500	5,000	10,000
**16**	**17**	**9**	**Bacillus/Mesobacillus/Cytobacillus**	500	500	500	50	500	10,000	1,000	5,000	5,000
**17**	**18**	**9**	**Bacillus/Mesobacillus/Cytobacillus**	500	1,000	500	50	100	> 10,000	2,500	5,000	5,000
**18**	**19**	**9**	**Bacillus/Mesobacillus/Cytobacillus**	2,500	> 5,000	1,000	50	10,000	> 10,000	10,000	10,000	10,000
**19**	**20**	**9**	**Bacillus/Mesobacillus/Cytobacillus**	500	> 5,000	500	50	2,500	> 10,000	5,000	5,000	>10,000
**20**	**21**	**9**	**Bacillus/Mesobacillus/Cytobacillus**	2,500	> 5,000	1,000	500	> 10,000	> 10,000	5,000	> 10,000	10,000
**21**	**23**	**9**	**Bacillus/Mesobacillus/Cytobacillus**	500	1,000	1,000	50	500	> 10,000	500	10,000	5,000
**22**	**24**	**6**	**Lysinibacillus/Sporosarcina**	500	500	500	50	100	> 10,000	500	2,500	5,000
**23**	**25**	**8**	**Bacillus/Peribacillus/Rossellomorea**	> 5,000	> 5,000	> 5,000	100	5,000	> 10,000	>10,000	> 10,000	>10,000
**24**	**26**	**8**	**Bacillus/Peribacillus/Rossellomorea**	1,000	> 5,000	1,000	100	2,500	> 10,000	5,000	10,000	10,000
**25**	**27**	**2**	**Ruegeria/Cribrihabitans**	2,500	500	500	50	100	> 10,000	2,500	5,000	5,000

**26**	**28**	**6** **Lysinibacillus/Sporosarcina**	1,000	500	500	50	2,500	> 10,000	1,000	5,000	5,000
**27**	**30**	**2** **Ruegeria/Cribrihabitans**	500	1,000	100	50	500	> 10,000	500	5,000	5,000
**28**	**31**	**2** **Ruegeria/Cribrihabitans**	2,500	> 5,000	500	50	1,000	> 10,000	1,000	10,000	10,000
**29**	**32**	**9** **Bacillus/Mesobacillus/Cytobacillus**	1,000	500	500	50	500	> 10,000	500	2,500	5,000
**30**	**33**	**2** **Ruegeria/Cribrihabitans**	500	2,500	500	50	500	> 10,000	5,000	5,000	5,000
**31**	**34**	**8** **Bacillus/Peribacillus/Rossellomorea**	2,500	> 5,000	500	50	5,000	> 10,000	5,000	10,000	>10,000
**32**	**35**	**2** **Ruegeria/Cribrihabitans**	1,000	500	500	50	500	> 10,000	5,000	5,000	>10,000
**33**	**36**	**9** **Bacillus/Mesobacillus/Cytobacillus**	2,500	> 5,000	5,000	50	> 10,000	> 10,000	5,000	> 10,000	>10,000
**34**	**37**	**2** **Ruegeria/Cribrihabitans**	500	2,500	500	50	100	> 10,000	2,500	2,500	5,000
**35**	**38**	**2** **Ruegeria/Cribrihabitans**	2,500	1,000	500	50	100	> 10,000	5,000	5,000	10,000
**36**	**39**	**2** **Ruegeria/Cribrihabitans**	1,000	1,000	500	50	500	10,000	500	10,000	10,000
**37**	**40**	**3** **Bacillus berkeleyi/decolorationis**	2,500	5,000	500	50	2,500	> 10,000	1,000	10,000	>10,000
**38**	**41**	**2** **Ruegeria/Cribrihabitans**	1,000	1,000	50	50	100	> 10,000	2,500	5,000	5,000
**39**	**42**	**9** **Bacillus/Mesobacillus/Cytobacillus**	500	1,000	500	50	100	> 10,000	500	2,500	2,500
**40**	**43**	**9** **Bacillus/Mesobacillus/Cytobacillus**	500	2,500	500	50	100	> 10,000	500	2,500	5,000

**41**	**45**	**8**<br>**Bacillus/Peribacillus/Rossellomorea**	1,000	1,000	50	50	100	> 10,000	500	2,500	10,000
**42**	**46**	**5**<br>**Bhargavaea**	500	500	500	50	100	> 10,000	500	2,500	2,500
**43**	**47**	**9**<br>**Bacillus/Mesobacillus/Cytobacillus**	2,500	> 5,000	500	100	> 10,000	> 10,000	>10,000	> 10,000	>10,000
**44**	**48**	**2**<br>**Ruegeria/Cribrihabitans**	500	500	500	50	1,000	> 10,000	500	2,500	5,000
**45**	**49**	**6**<br>**Lysinibacillus/Sporosarcina**	500	500	500	50	100	> 10,000	500	2,500	1,000
**46**	**50**	**9**<br>**Bacillus/Mesobacillus/Cytobacillus**	1,000	> 5,000	1,000	50	1,000	> 10,000	2,500	10,000	10,000
**47**	**52**	**9**<br>**Bacillus/Mesobacillus/Cytobacillus**	500	5,000	500	50	5,000	> 10,000	5,000	10,000	>10,000
**48**	**53**	**9**<br>**Bacillus/Mesobacillus/Cytobacillus**	> 5,000	500	500	50	500	> 10,000	500	5,000	5,000
**49**	**55**	**6**<br>**Lysinibacillus/Sporosarcina**	500	500	100	50	500	> 10,000	500	5,000	5,000
**50**	**56**	**9**<br>**Bacillus/Mesobacillus/Cytobacillus**	500	2,500	500	50	500	> 10,000	500	5,000	10,000
**51**	**57**	**4**<br>**Bacillus/Fictibacillus**	500	2,500	1,000	50	100	> 10,000	5,000	10,000	5,000
**52**	**58**	**9**<br>**Bacillus/Mesobacillus/Cytobacillus**	1,000	1,000	1,000	50	100	> 10,000	500	5,000	5,000
**53**	**61**	**2**<br>**Ruegeria/Cribrihabitans**	500	500	500	50	2,500	> 10,000	1,000	> 10,000	10,000
**54**	**62**	**8**<br>**Bacillus/Peribacillus/Rossellomorea**	2,500	500	500	50	500	ND	500	2,500	2,500
**55**	**63**	**6**<br>**Lysinibacillus/Sporosarcina**	1,000	1,000	500	50	500	> 10,000	5,000	5,000	2,500

**56**	**64**	**8**<br>**Bacillus/Peribacillus/Rossellomorea**	500	500	100	50	100	> 10,000	500	5,000	5,000
**57**	**65**	**4**<br>**Bacillus/Fictibacillus**	500	> 5,000	1,000	50	2,500	> 10,000	2,500	5,000	>10,000
**58**	**66**	**8**<br>**Bacillus/Peribacillus/Rossellomorea**	500	500	500	50	500	> 10,000	500	10,000	10,000
**59**	**67**	**8**<br>**Bacillus/Peribacillus/Rossellomorea**	5,000	5,000	500	50	1,000	> 10,000	5,000	> 10,000	5,000
**60**	**68**	**8**<br>**Bacillus/Peribacillus/Rossellomorea**	500	5,000	500	500	10,000	> 10,000	2,500	> 10,000	10,000
**61**	**69**	**7**<br>**Bacillus/Pseudoalkalibacillus/Alkalibacillus**	1,000	> 5,000	500	50	2,500	> 10,000	10,000	10,000	>10,000
**62**	**71**	**8**<br>**Bacillus/Peribacillus/Rossellomorea**	500	500	50	50	100	> 10,000	2,500	5,000	10,000
**63**	**72**	**9**<br>**Bacillus/Mesobacillus/Cytobacillus**	500	2,500	1,000	50	1,000	5,000	1,000	5,000	5,000
**64**	**73**	**7**<br>**Bacillus/ Pseudoalkalibacillus/ Alkalibacillus**	500	500	500	50	500	5,000	500	5,000	5,000
**65**	**75**	**1**<br>**Pseudoalteromonas**	500	1,000	500	50	500	1,000	1,000	5,000	10,000
**66**	**76**	**8**<br>**Bacillus/Peribacillus/Rossellomorea**	500	> 5,000	500	100	> 10,000	10,000	10,000	5,000	5,000
**67**	**78**	**2**<br>**Ruegeria/Cribrihabitans**	500	1,000	500	50	100	10,000	100	> 10,000	10,000
**68**	**81**	**8**<br>**Bacillus/Peribacillus/Rossellomorea**	> 5,000	> 5,000	2,500	50	> 10,000	> 10,000	>10,000	10,000	10,000
**69**	**82**	**9**<br>**Bacillus/Mesobacillus/Cytobacillus**	1,000	> 5,000	500	50	> 10,000	> 10,000	5,000	10,000	>10,000

**70**	**83**	**7**<br>**Bacillus/ Pseudoalkalibacillus/ Alkalibacillus**	500	500	500	50	100	> 10,000	500	2,500	2,500
**71**	**84**	**9**<br>**Bacillus/Mesobacillus/Cytobacillus**	1,000	5,000	500	50	5,000	> 10,000	10,000	5,000	10,000
**72**	**85**	**6**<br>**Lysinibacillus/Sporosarcina**	1,000	> 5,000	500	50	100	> 10,000	5,000	> 10,000	10,000
**73**	**87**	**1**<br>**Pseudoalteromonas**	500	500	500	50	1,000	100	1,000	5,000	5,000
**74**	**88**	**7**<br>**Bacillus/ Pseudoalkalibacillus/ Alkalibacillus**	500	500	500	50	2,500	> 10,000	2,500	5,000	5,000
